# Visual Equivalence and Amodal Completion in Cuttlefish

**DOI:** 10.3389/fphys.2017.00040

**Published:** 2017-02-06

**Authors:** I-Rong Lin, Chuan-Chin Chiao

**Affiliations:** ^1^Institute of Systems Neuroscience, National Tsing Hua UniversityHsinchu, Taiwan; ^2^Department of Life Science, National Tsing Hua UniversityHsinchu, Taiwan

**Keywords:** visual discrimination, visual perception, object recognition, size constancy, visual completion

## Abstract

Modern cephalopods are notably the most intelligent invertebrates and this is accompanied by keen vision. Despite extensive studies investigating the visual systems of cephalopods, little is known about their visual perception and object recognition. In the present study, we investigated the visual processing of the cuttlefish *Sepia pharaonis*, including visual equivalence and amodal completion. Cuttlefish were trained to discriminate images of shrimp and fish using the operant conditioning paradigm. After cuttlefish reached the learning criteria, a series of discrimination tasks were conducted. In the visual equivalence experiment, several transformed versions of the training images, such as images reduced in size, images reduced in contrast, sketches of the images, the contours of the images, and silhouettes of the images, were used. In the amodal completion experiment, partially occluded views of the original images were used. The results showed that cuttlefish were able to treat the training images of reduced size and sketches as the visual equivalence. Cuttlefish were also capable of recognizing partially occluded versions of the training image. Furthermore, individual differences in performance suggest that some cuttlefish may be able to recognize objects when visual information was partly removed. These findings support the hypothesis that the visual perception of cuttlefish involves both visual equivalence and amodal completion. The results from this research also provide insights into the visual processing mechanisms used by cephalopods.

## Introduction

Cephalopods possess the largest and most complex nervous systems in invertebrates (Nixon and Young, 2003). Their brains can be anatomically divided into 30–40 interconnected lobes that have similarities to the brain organization of vertebrates (Young and Boycott, 1971; Hochner, 2010). As highly visual animals, cephalopods exhibit a repertoire of sophisticated motor responses that are driven by their visual systems (Packard, 1972). Their keen vision assists them in executing a diverse series of complex behaviors such as camouflage body patterning and conspecific communication (Hanlon and Messenger, 1996). Therefore, it seems likely that vision has played an important role in shaping the evolution of cephalopod cognition (Darmaillacq et al., 2014). Although previous studies have demonstrated that cephalopods are capable of various types of visual discrimination, evidence indicating how the highly developed visual systems of cephalopods generate visual sensation and perception are lacking (Zylinski and Osorio, 2014).

Stimulus generalization is a fundamental cognitive ability that is characterized by an organism treating similar stimuli equivalently (Bruce et al., 2003). Basic generalization capacity is typically demonstrated by showing that animals with a learnt response to a given stimulus are able to transfer the established behavior to a novel stimulus that resembles the previous one (Shettleworth, 2009). Physical similarity between the perceived and stored information underlies stimulus generalization and therefore such transfer is both immediate and specific to a given stimulus (Marr, 2010). This adaptive response to new situations not only reduces the visual memory load of an organism, but also is likely to have the potential to increase the foraging success of the animal and to lower the threat from predators (Wynne and Udell, 2013).

Vertebrates and insects display high degrees of visual generalization (reviewed in Ghirlanda and Enquist, 2003; Horridge, 2009). For example, systematic studies using honeybees have shown that bees trained to recognize complex stimuli are able to transfer their choices to novel stimuli that preserved common features; these features include size, shape, orientation, pattern, and symmetry (Stach et al., 2004; Lehrer and Campan, 2005; Gross et al., 2009). However, visual generalization has seldom been investigated in cephalopods. Muntz (1961) studied interocular generalization in octopuses (*Octopus vulgaris*). Octopuses were trained to discriminate two visual stimuli using one eye, and then were tested using the untrained eye. Their results showed that the performance of octopuses in training had an impact on the degree of generalization. In a separate experiment, the same author also showed that octopuses trained to distinguish two complex shapes were able to transfer their responses to shapes that had different orientations to that of the original ones (Muntz, 1970). Similar to the aforementioned visual generalization, the ability of visual equivalence in cuttlefish was actually examined in the present study. Images are considered visually equivalent if they convey the same impressions of scene appearance, even if they are visibly different (Ramanarayanan et al., 2007).

Visual systems are known to engage in a process that allows active fill-in of absent details via connecting physically discontinuous image regions (Kanizsa, 1979; Michotte et al., 1991). This grouping mechanism allows the organism to perceive a complete rather than an incomplete form and is generally called “visual completion” (Bruce et al., 2003). This process has been divided into two types, modal and amodal. Visual completion by inducing a clear visual impression of a contrast border in an image region where there is no physical contrast border is known as “modal completion” (Snowden et al., 2012). The induced border is referred to as “illusory contour,” since it is not present in the physical stimulus. A classic example of modal completion is the Kanizsa triangle, which appears to most observers as a white triangle superimposed on three black discs (Kanizsa, 1979). On the other hand, visual completion by inducing a visual perception of a partially occluded object as an integral unity without generating any local contrast and illusory contours, which means that the perceived object has the same “mode” as the whole object, is known as amodal completion (Marr, 2010; Snowden et al., 2012). Thus, amodal interpolation of the likely form when there is an obscured region is based on the visible portions of the object.

The ability to carry out visual completion is ubiquitous in humans, and has been demonstrated in a number of other vertebrate taxa including non-human primates (Sato et al., 1997; Deruelle et al., 2000), rodents (Kanizsa et al., 1993), and fishes (Sovrano and Bisazza, 2008; Darmaillacq et al., 2011). Furthermore, honeybees are able to complete objects modally rather than amodally (Hateren et al., 1990; Horridge et al., 1992), which implies the possibility that other invertebrates may also be equipped with the ability to carry out visual completion. Recently, Zylinski et al. (2012) provided the first evidence of contour completion in cuttlefish (*Sepia officinalis*) by showing that cuttlefish respond with similar camouflage body patterns to either a whole visual stimulus or a fragmented visual stimulus.

In the present study, our goals were to examine the visual recognition capacities of one species of cuttlefish (*S. pharaonis*). We trained the cuttlefish to discriminate between two images using a newly developed behavioral paradigm. The images used in the study were artificial images of fish and shrimp. The performance of the cuttlefish thus allows us to evaluate their ability to carry out visual equivalence and amodal completion. Studying whether cuttlefish have similar visual processing mechanisms to their vertebrate counterparts, namely visual equivalence and completion, should increase greatly our understanding of convergent evolution in the context of animal visual processing.

## Materials and methods

### Animals

Twenty-one cuttlefish (*S. pharaonis*) from three different sources were used in the present study. Three animals formed Group A (cuttlefish A1–A3; mantle length, 3–5 cm) and were reared from eggs (trawled from the sea southwest of Taiwan near Tungkang and hatched in April 2011) at the National Museum of Marine Biology and Aquarium in Pingtung; these animals were transported to the National Tsing Hua University (NTHU) in Hsinchu for the experiments during June 2011. Ten animals formed Group B (cuttlefish B1–B10; mantle length, 5–12 cm) and these were also reared from eggs (collected by local fishermen fishing from Penghu and hatched in April 2011) at the National Penghu University of Science and Technology in Penghu; these animals were transported to the NTHU for the experiments during July 2011. Eight animals formed Group C (cuttlefish C1–C8; mantle length, 9–15 cm); these were sub-adult animals caught in northeastern of Taiwan near Yehliu, and were kept in the National Taiwan Ocean University at Keelung before being transported to the NTHU for experiments during February 2012. At NTHU the cuttlefish were housed individually in plastic tanks (depending on their mantle length; ML ≤ 4 cm: 33 cm × 23 cm × 24 cm, 4 cm ≤ ML ≤ 9 cm: 50 cm × 29 cm × 29 cm, and ML ≥ 9 cm: 78 cm × 50 cm × 30 cm), in two close-circulation aquariums (700 L each; water temperature 21 ~ 24°C). The cuttlefish were fed fish and shrimp twice daily and acclimated to the system at least 1 week prior to training. Training was started only when the cuttlefish showed signs of aggressive predation. All experiments were conducted in the home tanks of cuttlefish between 10 a.m. and 6 p.m. from July 2011 to May 2012. Five animals died during the training sessions and 16 cuttlefish completed the training. Among the trained animals, two died soon after the training and thus only 14 cuttlefish underwent testing (Table 1).

**Table 1:** **Number of discrimination training trials before reaching the learning criteria for each cuttlefish**.

**Cuttlefish**	**A1**	A2^*^	**B1**	**B2**	**B3**	**B4**	**B8**	**B9**	**B10**	**C1**	**C2**	**C4**	**C5**	**C6**	**C7**	C8^*^
# of trials	80	105	50	85	90	95	20	90	25	40	110	90	55	130	45	65

* *A2 and C8 died after training and did not take part in any of the later tests*.

### Apparatus

**Figure 1:**
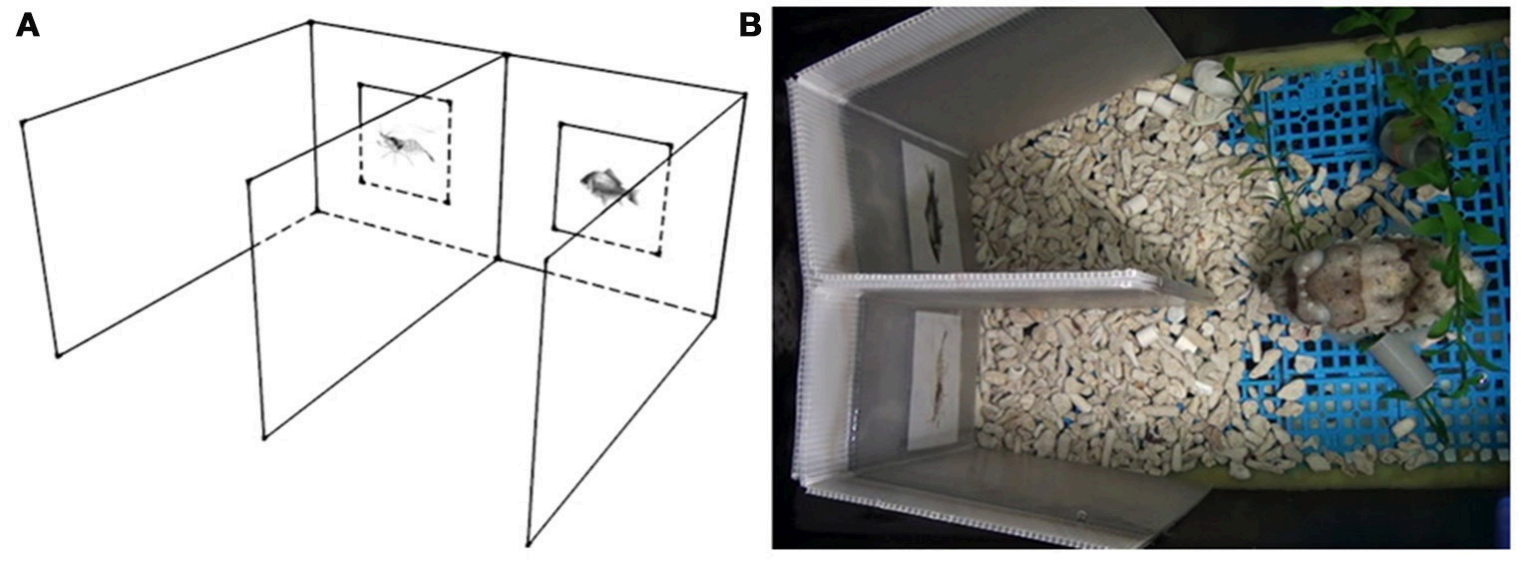
**The experimental setup. (A)** A schematic diagram of the apparatus from a side view. The apparatus was constructed to include two separate regions (the choosing areas) where the two visual stimuli were presented on the front wall. **(B)** Top view of the apparatus in the home tank of the cuttlefish. Cuttlefish at the choice point could see both stimuli simultaneously.

The apparatus was constructed of white corrugated plastic sheets and included two separate regions (the choosing areas), where two different visual stimuli were presented on the front walls at a height of 5 cm above ground (Figure 1). The two lateral walls were flexible and could be swung toward or away from the central divider. This design allowed the visual stimuli to be covered before putting the apparatus in the tank for training or testing. The visual stimuli were revealed by slowly swinging out lateral walls for viewing only after the cuttlefish had settled down. This also ensured that the cuttlefish saw both visual stimuli simultaneously at the start of each trial. Both visual stimuli were illuminated equally during the experiment, though the central divider may sometimes cause slight shadows on visual stimuli.

### Visual stimuli

**Figure 2:**
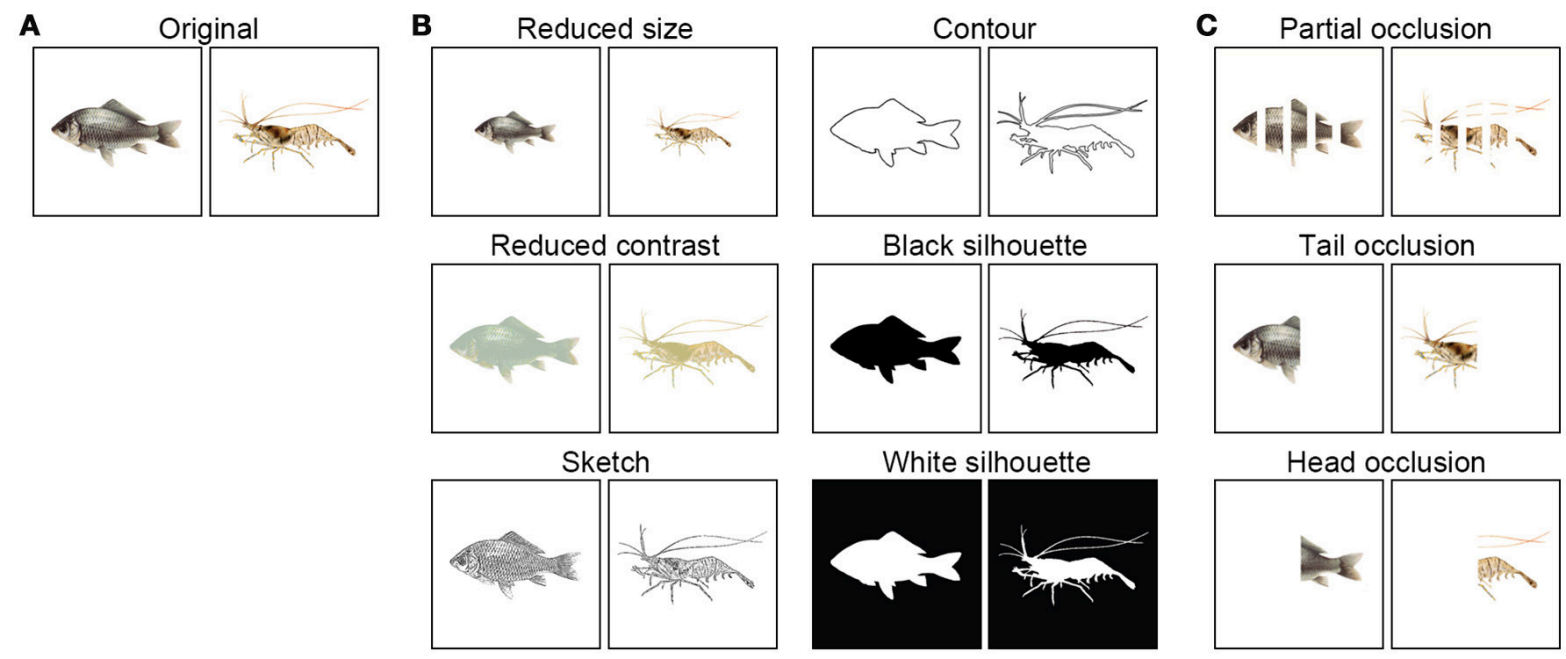
**Visual stimuli used in the present study. (A)** Fish and shrimp images were utilized for the discrimination training. **(B)** Six versions of the original images were used in the generalization tasks. **(C)** Three variations of the original images were used for the amodal completion tasks.

Pictures of fish and shrimp (length, 6.5 cm) were downloaded from the internet (Figure 2A). To investigate whether cuttlefish are equipped with the object recognition abilities to carry out visual equivalence and amodal completion tasks, several sets of paired images were modified from the originals using a graphic editing program (Ulead PhotoImpact X3). The reason that the images of fish and shrimp were chosen in the present study, instead of the simpler figures such as square and circle, is that cuttlefish were difficult to train to associate an abstractive stimulus with a reward. Since the cuttlefish were fed both fish and shrimp, it is unlikely that they have strong prey preferences. Furthermore, either a fish image or a shrimp image was randomly assigned to each individual cuttlefish before training (see below), thus the bias of their choice and learning ability due to the experience was reduced. To make the reduced size images, the original images of the fish and shrimp were resized to 60% of their original size (Figure 2B, up-left). To reduce the contrast of the fish and shrimp, the image contrast was adjusted to 50% of the original contrast (Figure 2B, mid-left). To create sketches of fish and shrimp, the sharpening effect of graphic editing program was used first to enhance edges and the image was thresholded to create a binary version (Figure 2B, bottom-left). To generate the contoured images, the outlines of animals were traced individually by hand (Figure 2B, up-right). To make the black silhouettes, the contoured region was filled with black (Figure 2B, mid-right). To make the white silhouettes, the contrast polarity was reversed from black to white (Figure 2B, bottom-right). The selectively occluded (amputated) images were generated by covering specific areas of the animals with white stripes (Figure 2C). These images consisted of partial occlusion (25% of the body covered by four stripes), tail occlusion (the posterior half covered), and head occlusion (the anterior half covered). The images were printed using a high quality laser printer (HP LaserJet P2055), then cut to give an 8.2 × 8.2 cm square with each pattern in center. Finally the images were laminated to make them waterproof.

### Discrimination training

The cuttlefish were trained to discriminate images of fish and shrimp (Figure 2A) using the operant conditioning paradigm. The goal is to train cuttlefish to strike reliably either a fish or a shrimp image with their tentacles. The reward image, a fish image or a shrimp image, was randomly assigned to each individual cuttlefish before training. Since cuttlefish do not naturally strike an object or image, the food (frozen shrimp) was initially presented in front of the reward image to draw animal's attention (i.e., the cuttlefish turned toward the reward image and showed convergence eye movement). During the visual attack of the cuttlefish (*S. officinalis*), it has been reported that attention is the first phase of the response (Messenger, 1968). Specifically, in attention there are color changes and movements of the eyes and head. The whole animal turns so that the prey comes to lie on a forward extension of the body axis. As soon as cuttlefish showed a sign of attention to the presentation of visual stimuli, swam into the reward image area, or carried out a strike on the image within 60 s, the food was delivered as a reward to motivate cuttlefish continuously performing this discrimination task. Each trial lasted 3 min, or until the cuttlefish made a correct choice. Each cuttlefish received five training trials per day. The position of the reward image was randomly assigned to the left or right in each trial. The discrimination training was considered complete only when cuttlefish achieved the learning criterion, which was an 80% correct response (that is choosing the reward image in 8 out of 10 trials over 2 consecutive days). To ensure the cuttlefish were able to discriminate the reward image from the non-reward image, after the training session a discrimination test was conducted. During this test the non-reward image was replaced by a novel image, such as a crab image, and the discrimination ability of each cuttlefish was then assessed again (data not shown).

### Transfer tests

A transfer test was conducted after animals passed the discrimination test to examine if cuttlefish are capable of visual equivalence and amodal completion. Each animal received 10 trials (five trials each day for two consecutive days) in a transfer test to retain the motivation of cuttlefish in performing the task. The position of the trained image was randomly assigned to the left or right in each trial, and the experimenter was not blind to the assignment of the previously rewarded image to each cuttlefish. To keep cuttlefish paying attention to the experimental apparatus, reward was offered for every correct response. If cuttlefish chose the previously non-reward image or did not respond at all in 5 min, the experimental apparatus was removed immediately, and the trial started again. To eliminate the effect of reinforcement and extinction, the image was covered during food delivery or before removing the apparatus. Between different transfer tests, an inter-test training session was held for cuttlefish to reinforce the conditioned response. Only when cuttlefish achieved the learning criterion of 80% correct response again, then a different transfer test was conducted. There were nine transfer tests (six for visual equivalence and three for amodal completion) that took place during the present study (Figures 2B,C).

### Scoring

**Figure 3:**
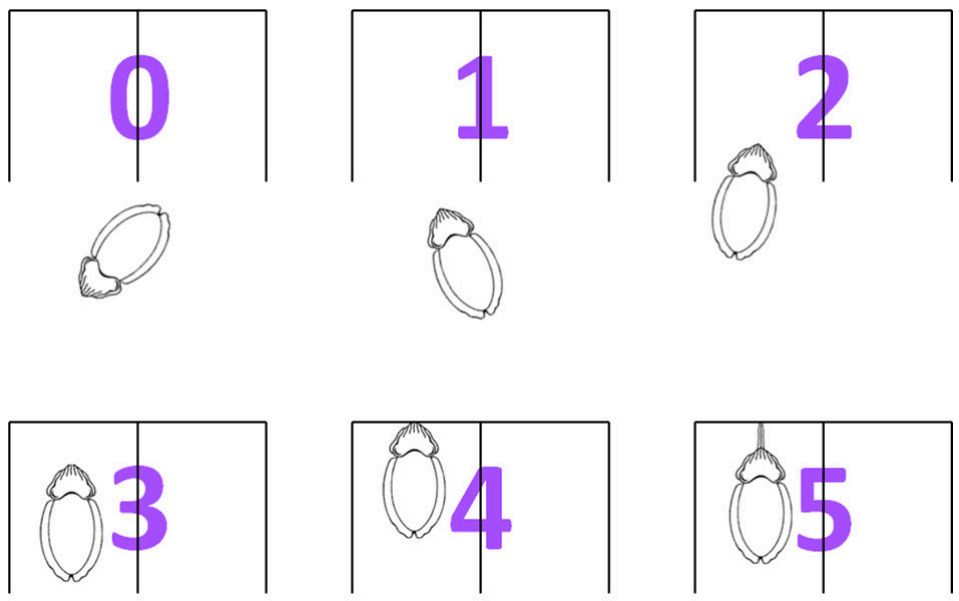
**The six levels of cuttlefish choosing response**. The cuttlefish response in each task was graded into six scores: (0) no attention on the apparatus, (1) stared at the figure with continuous attention (i.e., the whole animal turned so that the image came to lie on a forward extension of the body axis, subtending equal angles to the two eyes) for 1 min without entering the reward area, (2) stared at the figure with continuous attention <1 min and enter the reward area, (3) stayed in the reward area at a short distance from the previously rewarded figure for 30 s, (4) touched the previously rewarded figure with its arms, (5) struck the previously rewarded figure with its tentacles.

The cuttlefish response in each task was graded at six levels (Figure 3): (0) no attention paid to the apparatus, (1) stared at the image with continuous attention (i.e., the whole animal turned so that the image came to lie on a forward extension of the body axis, subtending equal angles to the two eyes) for 1 min without entering the reward area, (2) stared at the image with continuous attention <1 min and entered the reward area, (3) stay in the reward area at a short distance from the previously rewarded image for 30 s, (4) touched the previously rewarded image with its arms, (5) struck at the previously rewarded image with its tentacles. Cuttlefish were considered making a correct choice when they showed any of the score above zero responses in a trial.

### Data analysis

The binomial test was used to examine the statistical significance of the difference between the numbers of correct choices and incorrect choices for each animal over the nine transfer tests by comparing with the expected frequency of 50%. The score for each trial was normalized to the strongest response determined in the earlier discrimination training for each cuttlefish. The one-tailed Wilcoxon Signed Ranks test of the normalized scores was used to assess the choosing tendency of each animal over the nine transfer tests by comparing with the expected normalized score of zero. In addition, the one-tailed Wilcoxon Signed Ranks test was used to determine the choice tendency of all cuttlefish by analyzing the correct response percentages and the normalized scores obtained in each transfer task. All statistical analysis was conducted using SPSS.

## Results

**Figure 4:**
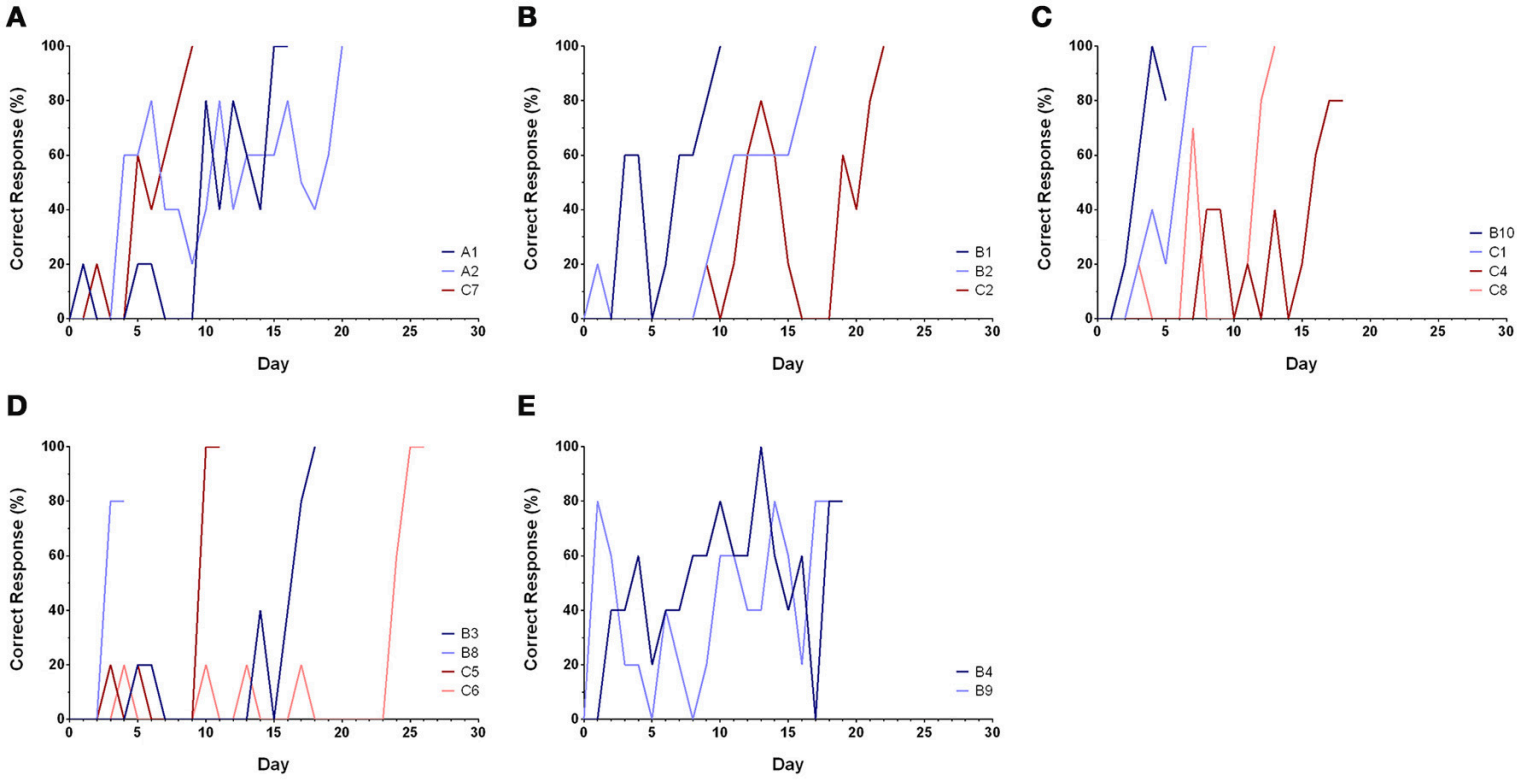
**Learning curves of cuttlefish in the discrimination training. (A–C)** Learning behaviors of most cuttlefish were a typical S-shape, showing a relatively gradual improvement. **(D)** Few correct responses initially and followed by an extremely rapid improvement. **(E)** Early fast learning and followed by a slow improvement. It is apparent that some animals did not respond to the reward image at all in the first few days (i.e., scored 0 point) or chose the non-reward image at the beginning of the training.

Sixteen of the 21 cuttlefish finished discrimination training (Table 1), while five died during training and two died immediately after training (A2 and C8). Among these 16 trained cuttlefish, two animals (B8 and B10) reached the learning criteria in <25 trials, and another three animals (B1, C1, and C7) reached the learning criteria in <50 trials. These animals appeared to be faster learners. Discrimination learning was confirmed when the percentage of correct responses of the cuttlefish rose from below chance (50% correct) to a success rate ranging from 80 to 100% (Figure 4). After completion of discrimination training, all cuttlefish reached the response level of 5, except A1 and B9 which only attained the response level of 3 (see Supplementary Information). The performance of the cuttlefish improved over time and the learning curves for most of the cuttlefish were S-shape, though some animals showed few correct responses initially and followed by an extremely rapid improvement (Figure 4D). All data including the results from training sessions and transfer tasks (below) were provided as the Supplementary Information.

### Visual equivalence

**Figure 5:**
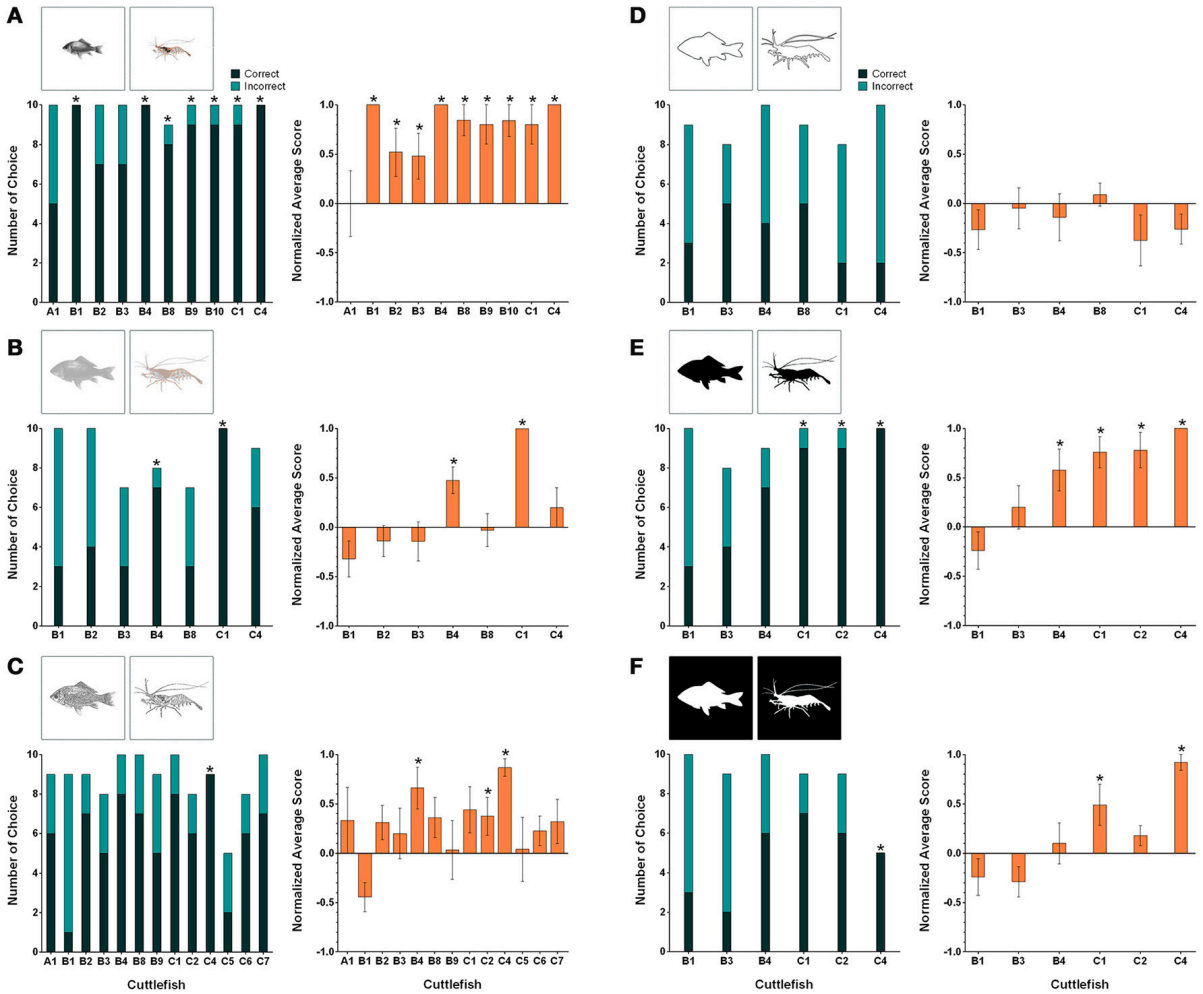
**The results for individual cuttlefish during the six visual equivalence tasks: (A)** Reduced size, **(B)** Reduced contrast, **(C)** Sketch, **(D)** Contour, **(E)** Black silhouette, and **(F)** White silhouette. The left panels show the correct/incorrect number of choices made by individual cuttlefish during these tasks. The correct response was determined when cuttlefish showed any of the score above zero responses in a trial (see Section Materials and Methods for scoring). Asterisks indicate statistical significance for the correct choice (*p* < 0.05). The right panels show the average normalized scores of the individual cuttlefish for the same tasks. The scores were normalized against the strongest response in the training. Asterisks indicate a significant tendency toward the reward figure. Note that cuttlefish B1 was significant for the incorrect choice and the tendency toward the non-reward figure in the sketch task, but asterisks were not labeled. Error bars are SEM.

When the transfer task involving the original fish and shrimp images being changed to reduce-scale images was carried out, the percentages of correct responses for seven cuttlefish (B1, B4, B8, B9, B10, C1, and C4) were higher than 80% (Figure 5A, left panel). For these animals, the numbers of correct choices were significant higher than those of the incorrect choices (binomial test, see Table 2). In terms of the cuttlefish average normalized responses, the scores of nine animals (B1, B2, B3, B4, B8, B9, B10, C1, and C4) were above 0.4 and seven of them were even higher than 0.7 (Figure 5A, right panel). Interestingly, cuttlefish B1, B4, and C4 obtained a score of +5 for all test trials. The same nine cuttlefish also showed a significant tendency to choose the rewarded images (Wilcoxon signed-rank test, see Table 2).

**Table 2:** **Statistical results for each individual cuttlefish across all tasks**.

**Testing stimuli**	**Statistical analysis**	**Cuttlefish**														
			**A1**	**B1**	**B2**	**B3**	**B4**	**B8**	**B9**	**B10**	**C1**	**C2**	**C4**	**C5**	**C6**	**C7**
Reduced size	Binomial test	P	0.623	0.001^*^	0.172	0.172	0.001^*^	0.020^*^	0.011^*^	0.011^*^	0.011^*^	–	0.001^*^	–	–	–
	Wilcoxon signed-rank test	W	0	55	43	39	55	43	44	53	44	–	55	–	–	–
		Z	0.000	−3.162	−2.282	−2.040	−3.162	−2.758	−2.530	−2.940	−2.530	–	−3.162	–	–	–
		P	0.500	0.001^*^	0.011^*^	0.021^*^	0.001^*^	0.003^*^	0.006^*^	0.002^*^	0.006^*^	–	0.001^*^	–	–	–
Low contrast	Binomial test	P	–	0.172	0.377	0.500	0.035^*^	0.500	–	–	0.001^*^	–	0.254	–	–	–
	Wilcoxon signed-rank test	W	–	−31	−17	−10	33	−2	–	–	55	–	15	–	–	–
		Z	–	−1.596	−0.889	−0.862	−2.328	−0.171	–	–	−3.162	–	−1.000	–	–	–
		P	–	0.056	0.187	0.195	0.010^*^	0.432	–	–	0.001^*^	–	0.159	–	–	–
Sketch	Binomial test	P	0.254	0.020^*^	0.090	0.363	0.055	0.172	0.500	–	0.055	0.145	0.002^*^	0.500	0.145	0.172
	Wilcoxon signed-rank test	W	15	−35	23	13	49	32	1	–	29	24	45	1	19	25
		Z	−1.000	−2.090	−1.475	−0.933	−2.595	−1.638	−0.064	–	−1.499	−1.703	−2.810	−0.137	−1.354	−1.287
		P	0.159	0.019^*^	0.070	0.176	0.005^*^	0.051	0.475	–	0.067	0.044^*^	0.003^*^	0.446	0.088	0.099
Contour	Binomial test	P	–	0.254	–	0.363	0.377	0.500	–	–	0.145	–	0.055	–	–	–
	Wilcoxon signed-rank test	W	–	−25	–	−5	−17	13	–	–	−16	–	−27	–	–	–
		Z	–	−1.530	–	−0.352	−0.900	−0.787	–	–	−1.206	–	−1.430	–	–	–
		P	–	0.063	–	0.363	0.184	0.216	–	–	0.114	–	0.077	–	–	–
Black silhouette	Binomial test	P	–	0.172	–	0.637	0.090	–	–	–	0.011^*^	0.011^*^	0.001^*^	–	–	–
	Wilcoxon signed-rank test	W	–	−22	–	13	37	–	–	–	52	51	55	–	–	–
		Z	–	−1.135	–	−0.923	−2.239	–	–	–	−2.716	−2.754	−3.162	–	–	–
		P	–	0.129	–	0.178	0.013^*^	–	–	–	0.004^*^	0.003^*^	0.001^*^	–	–	–
White silhouette	Binomial test	P	–	0.172	–	0.090	0.377	–	–	–	0.090	0.254	0.031^*^	–	–	–
	Wilcoxon signed-rank test	W	–	−22	–	−27	6	–	–	–	37	27	15	–	–	–
		Z	–	−1.188	–	−1.616	−0.318	–	–	–	−2.239	−1.634	−2.121	–	–	–
		P	–	0.118	–	0.053	0.376	–	–	–	0.013^*^	0.051	0.017^*^	–	–	–
Partial occlusion	Binomial test	P	–	0.020^*^	–	0.254	0.623	–	–	–	0.637	0.500	0.035^*^	–	0.016^*^	0.002^*^
	Wilcoxon signed-rank test	W	–	41	–	27	12	–	–	–	4	−1	31	−	21	45
		Z	–	−2.459	–	−1.616	−0.624	–	–	–	−0.302	−0.137	−2.226	−	−2.232	−3
		P	–	0.007^*^	–	0.053	0.267	–	–	–	0.382	0.446	0.013^*^	–	0.013^*^	0.002^*^
Posteriorly occlusion	Binomial test	P	–	–	–	–	–	–	–	–	0.055	–	0.001^*^	–	0.011^*^	0.001^*^
	Wilcoxon signed-rank test	W	–	–	–	–	–	–	–	–	33	–	55	–	44	55
		Z	–	–	–	–	–	–	–	–	−1.897	–	−3.162	–	−2.530	−3.162
		P	–	–	–	–	–	–	–	–	0.029^*^	–	0.001^*^	–	0.006^*^	0.001^*^
Anteriorly occlusion	Binomial test	P	–	–	–	–	–	–	–	–	0.377	–	0.172	–	0.055	0.254
	Wilcoxon signed-rank test	W	–	–	–	–	–	–	–	–	13	–	30	–	40	19
		Z	–	–	–	–	–	–	–	–	−0.690	–	−1.576	–	−2.160	−1.150
		P	–	–	–	–	–	–	–	–	0.245	–	0.058	–	0.016^*^	0.125

*The binomial test was used to examine the correct responses from the individual animals for each task (p, p-value). The one-tailed Wilcoxon signed-rank test was applied to analyze the normalized scores obtained from the individual animals for each task (W, test statistic; Z, the Z statistic; p, p-value). Asterisks indicate statistical significance*.

Using the low contrast version of the original images as stimuli, two cuttlefish (B4 and C1) exhibited 80% correct responses (Figure 5B, left panel). The correct choices made by these two animals were significant higher than the incorrect choices (binomial test, see Table 2). The average normalized scores were 0.475 and 1 (i.e., got +5 scores for all 10 test trials), respectively (Figure 5B, right panel). A significant tendency to target the rewarded image was also found (Wilcoxon signed-rank test, see Table 2).

When the initial images were replaced by sketches, the percentages of correct responses of three animals (B4, C1, and C4) reached 80% (Figure 5C, left panel). The correct choices made by cuttlefish C4 were significant higher than its incorrect choices (binomial test, see Table 2). Note that cuttlefish B1 preferred the non-reward image significantly (*p* = 0.02) for no obvious reason. In addition, the average normalized scores of two cuttlefish B4 and C4 were higher than 0.5 (Figure 5C, right panel). However, cuttlefish B4, C2, and C4 showed a significant tendency to choose the rewarded image (Wilcoxon signed-rank test, see Table 2).

The performance of all six cuttlefish toward the contoured original image was poor. The percentages of correct responses were lower than the 50% chance level (Figure 5D, left panel). None of these animals ever obtained a +5 score in a test trial and the average normalized scores were all <0.1 (Figure 5D, right panel). No significant trend was found (Wilcoxon signed-rank test, see Table 2).

When the stimuli were black silhouettes of original images on a white background, the percentages of correct choice of three cuttlefish (C1, C2, and C4) were higher than 80% (Figure 5E, left panel). The correct choices made by these three animals were significant higher than the incorrect choices (binomial test, see Table 2). The average normalized scores of four cuttlefish (B4, C1, C2, and C4) were higher than 0.5 (Figure 5E, right panel), and they also showed a significant tendency to choose the rewarded image (Wilcoxon signed-rank test, see Table 2).

In the case of white silhouettes of the original images on a black background, the percentages of correct choice were above 50% for four cuttlefish (B4, C1, C2, and C4; Figure 5F, left panel). However, only cuttlefish C4 made five correct choices and five undetermined responses and thus with this animal the number of correct choices was significant higher than its incorrect choices (binomial test, see Table 2). The average normalized scores of cuttlefish C1 and C4 were 0.489 and 0.920, respectively (Figure 5F, right panel), and a significant tendency toward the rewarded images was also found (Wilcoxon signed-rank test, see Table 2).

**Figure 6:**
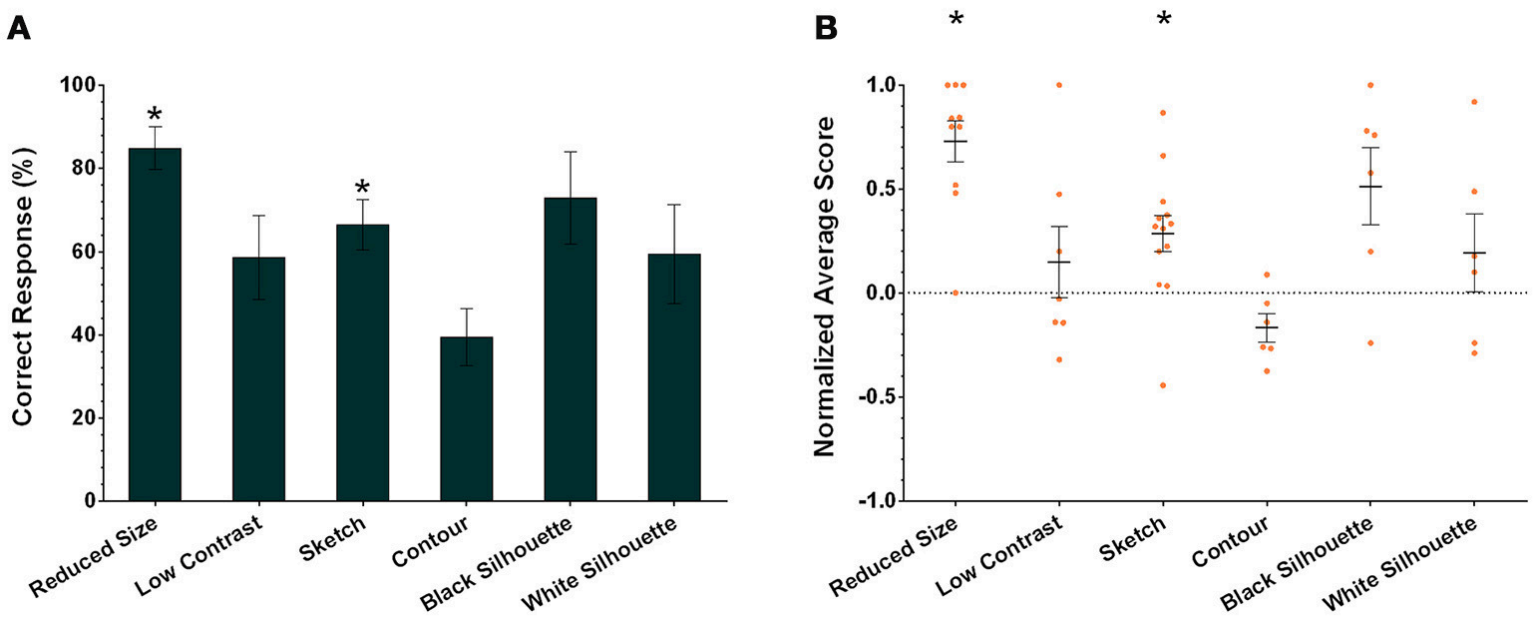
**The population results of cuttlefish in the six visual equivalence tasks. (A)** Average correct response percentages of all cuttlefish in each task. **(B)** Average normalized scores of all animals in each task. Orange dots represent individual data. *N* = 10, 7, 13, 6, 6, and 6 for reduced size, reduced contrast, sketch, contour, black silhouette, and white silhouette, respectively. Asterisks indicate statistical significance (*p* < 0.05). Error bars are SEM.

In addition to assessing the responses of individual cuttlefish, we also consider the group performance for each task. Cuttlefish tended to respond to the rewarded images in the visual equivalence tasks when the images were reduced in size and sketches (Figure 6A; one-tailed Wilcoxon signed-rank test, see Table 3). Similarly, taking the strength of the responses into account, these animals also exhibited strong responses in tasks when the images were reduced in size and sketches (Figure 6B; one-tailed Wilcoxon signed-rank test, see Table 3). Even though the individual responses had at least one or more animals showed the statistical significance in five of six tasks (except in the contour test), due to the small sample size in some experiments, the population results only supported cuttlefish's capacity in two of six visual equivalence tasks (reduced size and sketch).

**Table 3:** **Statistical results for all cuttlefish across nine tasks**.

**Testing stimuli**	**Statistical analysis**		**Population**	
			**Correct response percentage**	**Normalized average score**
Reduced size	Wilcoxon signed-rank test	W	45	45
		Z	−2.687	−2.687
		P	0.007^*^	0.007^*^
Low contrast	Wilcoxon signed-rank test	W	6	6
		Z	−0.508	−0.508
		P	0.611	0.611
Sketch	Wilcoxon signed-rank test	W	63	63
		Z	−2.203	−2.203
		P	0.028^*^	0.028^*^
Contour	Wilcoxon signed-rank test	W	−13	−13
		Z	−1.363	−1.363
		P	0.173	0.173
Black silhouette	Wilcoxon signed-rank test	W	13	13
		Z	−1.761	−1.761
		P	0.078	0.078
White silhouette	Wilcoxon signed-rank test	W	6	6
		Z	−0.631	−0.631
		P	0.528	0.528
Partial occlusion	Wilcoxon signed-rank test	W	21	21
		Z	−2.207	−2.207
		P	0.027^*^	0.027^*^
Posteriorly occlusion	Wilcoxon signed-rank test	W	10	10
		Z	−1.841	−1.841
		P	0.066	0.066
Anteriorly occlusion	Wilcoxon signed-rank test	W	10	10
		Z	−1.826	−1.826
		P	0.068	0.068

*The one-tailed Wilcoxon signed-rank test was used to determine the choices made by all cuttlefish (W, test statistic; Z, the Z statistic; p, p-value), and the same test was also applied to analyze the normalized scores obtained from all animals in each task. Asterisks indicate statistical significance*.

### Amodal completion

**Figure 7:**
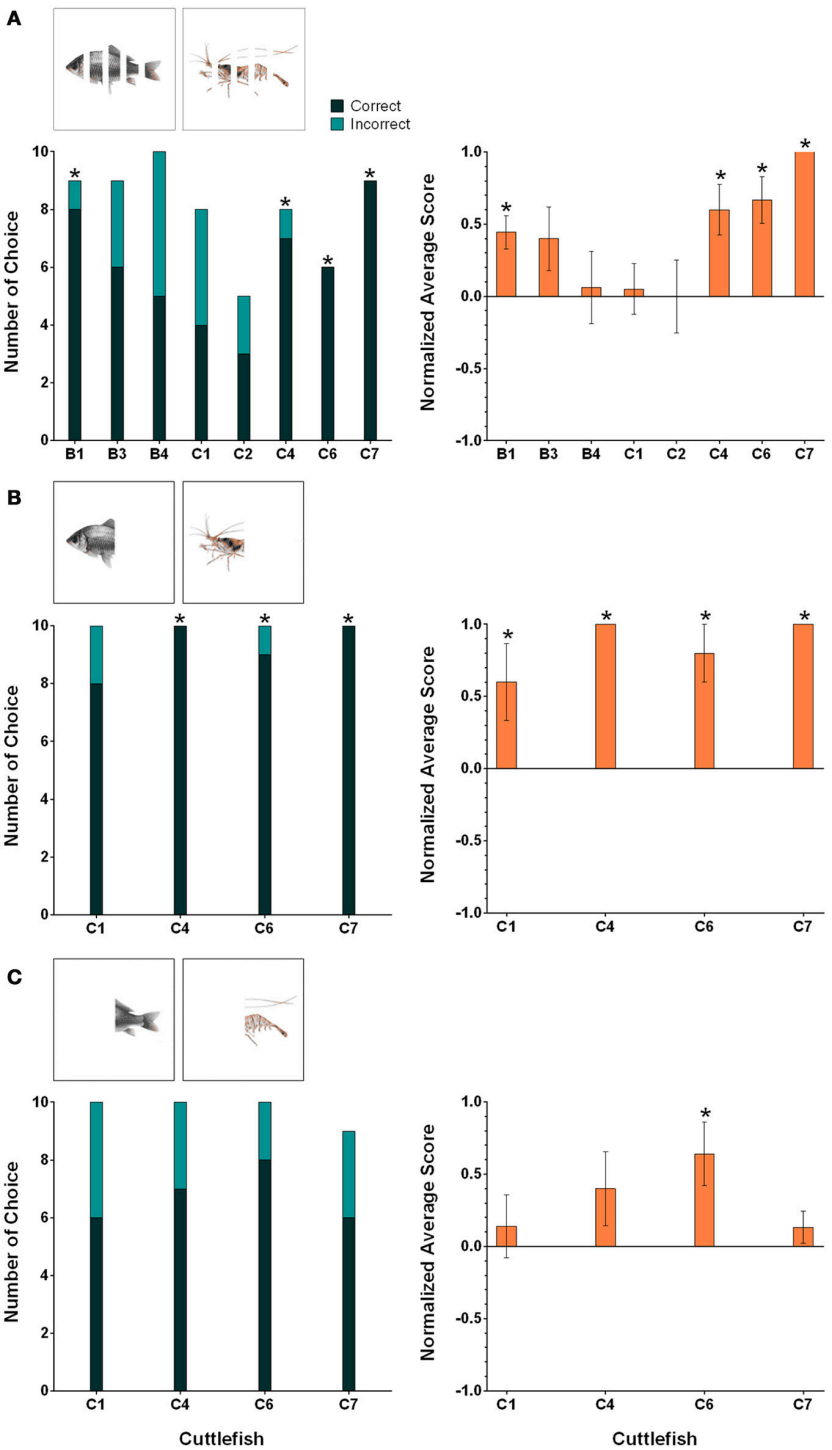
**The results for individual cuttlefish during the three amodal completion tasks: (A)** Partial occlusion, **(B)** Tail occlusion, and **(C)** Head occlusion. The left panels show the correct/incorrect number of choices made by individual cuttlefish during these amodal completion tasks. The correct response was determined when cuttlefish showed any of the score above zero responses in a trial (see Section Materials and Methods for scoring). Asterisks indicate statistical significance for the correct choice (*p* < 0.05). The right panels show the average normalized scores of the individual cuttlefish for the same tasks. The scores were normalized against the strongest response in the training. Asterisks indicate a significant tendency toward the rewarded figure. Error bars are SEM.

During the first amodal completion task, the fish and shrimp images were partially occluded by four 0.4 cm white stripes (25% of the body covered by four stripes) and under these conditions, the percentages of correct choices of four cuttlefish (B1, C4, C6, and C7) were above 80% (Figure 7A, left panel). For these four animals, the numbers of correct choices were significant higher than those of incorrect choices (binomial test, see Table 2). The average normalized scores of these four animals were higher than 0.4 (Figure 7A, right panel). A significant tendency toward the rewarded image was found for these animals (Wilcoxon signed-rank test, see Table 2).

In the second amodal completion task, fish and shrimp images were posteriorly occluded (head visible) and the results showed that the percentages of correct responses of all four cuttlefish were higher than 80% (Figure 7B, left panel). The correct choices made by three animals (C4, C6, and C7) were significantly higher than their incorrect choices (binomial test, see Table 2). The average normalized scores of all cuttlefish were above 0.6 (Figure 7B, right panel). In addition, cuttlefish C4 and C7 obtained +5 scores for all 10 test trials. All four animals had a significant tendency to choose the rewarded images (Wilcoxon signed-rank test, see Table 2).

During the final amodal completion task, the fish and shrimp images were anteriorly occluded (tail visible). In this part of the study, the percentage of correct choice of only one cuttlefish C6 was above 80% (Figure 7C, left panel), but no statistical significant was found (binomial test, see Table 2). Among these subjects, the average normalized score of cuttlefish C6 was 0.64 (Figure 7C, right panel), and it showed a significant tendency toward the reward image (Wilcoxon signed-rank test, see Table 2).

**Figure 8:**
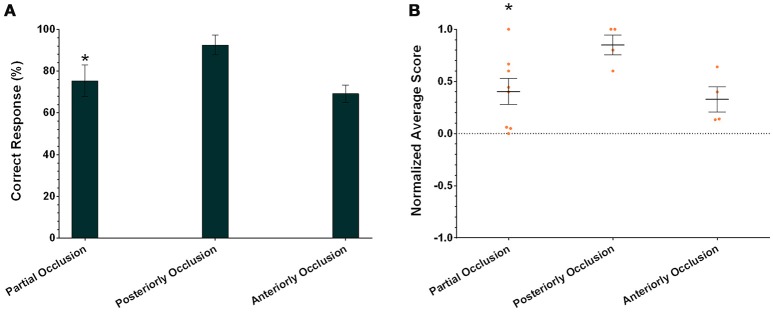
**The population results of cuttlefish in the three amodal completion tasks. (A)** Average correct response percentages of all cuttlefish in each task. **(B)** Average normalized scores of all animals in each task. Orange dots represent individual data. *N* = 8, 4, and 4 for partial occlusion, tail occlusion, and head occlusion, respectively. Asterisks indicate statistical significance (*p* < 0.05). Error bars are SEM.

In addition to assessing the responses of individual cuttlefish, we also consider the group performance for each task. Due to the small sample size in the present study, cuttlefish tended to respond to the rewarded images in only the partial occlusion task (Figure 8A; one-tailed Wilcoxon signed-rank test, see Table 3). Even taking the strength of the responses into account, cuttlefish still exhibited strong responses only in the partial occlusion task (Figure 8B; one-tailed Wilcoxon signed-rank test, see Table 3). However, it is apparent that the individual responses had at least one or more animals showed the statistical significance in all three tasks, thus although the population results only supported cuttlefish's capacity in the partial occlusion task, it is likely that cuttlefish are also capable of amodal completion at least in the posterior occlusion task.

## Discussion

### Visual association learning in cuttlefish

Although some cuttlefish took a significant longer time to learn the association between the visual stimulus and the reward, once they had learnt, they could be tested using a range of different visual perception tasks. More importantly, the time cuttlefish spent learning (Figure 4) appears to be independent of their performance in these transfer tests. This suggests that there is variability between individual cuttlefish regarding visual association learning and reliability when carrying out visual perception tasks.

In addition to striking the rewarded target, some other behavioral features were observed during the training and these might be useful when assessing cuttlefish learning. For example, cuttlefish tended to approach the target image with a “stop-and-go” or stealth-type locomotion while raising and waving their first pair of arms in front of the target image and then changing their skin coloration on recognizing the target image. These signs suggest that the cuttlefish is paying attention to the target image or at least is able to recognize the visual stimulus during both training and testing. Interestingly, we also found that all cuttlefish responded to the reward image with a tentacle strike initially, but after a few trials, some animals begin to grab the images with their arms instead. This behavioral shift in their foraging strategy may result from pain when the tentacles strike (Messenger, 1973) and is another indication of learning by visual association.

### Object perception and visual equivalence

Species that live in rich and diverse natural environments need visual systems that work hard in order to process and organize the very large amount of visual information that is received by the organism's eyes (Land and Nilsson, 2012; Cronin et al., 2014). Visual generalization and equivalence is a fundamental ability that helps an individual to deal with similar visual events and helps the individual to make consistent responses without repeated information processing (Bruce et al., 2003). The ability to carry out generalization is found in a wide range of animals and is indispensable to survival in a constantly changing environment (Marr, 2010). This is because what has been learned from a limited experience is unlikely to recur in an identical form again. For example, bee foragers need to identify appropriate flowers regardless of their orientation, shape, color, illumination, etc. and therefore generalization of these features assists their forage success (Horridge, 2009).

In the present study, the strongest evidence of visual equivalence is presented by the data from the task with reduced size images, in which nine out of ten cuttlefish gave significant responses to the correct images. This result indicates that cuttlefish exhibit a highly degree of visual equivalence for size and it is not hard to understand why this is true. Specifically, there are abundant details of the prey preserved in the images and evolutionarily it seems likely that cuttlefish will want to know a larger prey and a smaller prey are both prey. Similar size equivalence has been demonstrated widely in vertebrates (Guttman and Kalish, 1956; Jenkins et al., 1958; Ewert, 1980; Dougherty and Lewis, 1991) and insects (Tinbergen et al., 1942). For instance, rats trained to open a door in the center of a white circle was able to transfer their responses with respect to opening doors in circles of a variety of different sizes. The ability to make a consistent judgment with respect to similar objects independent of its physical size resembles the concept of size constancy, which refers to the invariant judgment that occur with a particular object regardless of their size on the retina (Bruce et al., 2003; Marr, 2010; Snowden et al., 2012). Size constancy has been demonstrated in both vertebrates (Pastore, 1958; Lombardi and Delius, 1990) and insects (Jacobs-Jessen, 1959). For example, goldfish trained to discriminate between two similar objects of different sizes were able to exhibit successful discrimination when these objects are placed at different distances from the fish so as to subtend the same visual angle on the retina (Douglas et al., 1988). In the experiment using cephalopods, cuttlefish (*S. officinalis*) were trained to discriminate between squares of different sizes and were found to show size constancy (Messenger, 1977).

Visual generalization is not merely restricted to a single feature. Multi-feature generalization, which involves complex patterns, has been extensively studied in honeybees. Bees can be trained to discriminate circular patterns with differently oriented gratings in four quadrants and were able to transfer their choices to a corresponding simplified situation (Stach et al., 2004). Moreover, the degree of transfer was found to be dependent on the training length and prolonging the training length led to a promotion of both the generalization level and the discrimination strategy shift (Stach and Giurfa, 2005).

Well-experienced bees tend to extract only the minimum necessary information needed for discrimination since they cannot distinguish the original pattern from the simplified pattern. It has also been shown that the processing strategies involved in visual recognition include a shift from the elemental to the global as the trial numbers further increase, and this shift could decrease the bee's performance in recognizing the original image (Giurfa et al., 2003). In the present study, we found that tentacle strikes mainly occurred during the first one to two trials of the task with the sketched images, and the performance of some animals declined during the subsequent trials (see Supplementary Information). If we consider this in terms of the visual recognition strategy shift that occurs with bees, we suggest that cuttlefish use a similar strategy change for visual recognition. That is, cuttlefish might initially be concerned about the detailed information available, including structures, textures, and outlines, but subsequently they acquire a global view of the sketched image, the integral style of the image held by the cuttlefish has now become very different from the original image.

Generalization is a process that involves feature extraction and therefore systematic studies on generalization should be able to provide a suitable way of identifying the visual cues utilized during visual recognition. Research on honeybee vision has a long tradition and generalization does indeed play an important role in understanding how the visual perception of bees operates (Ronacher, 1998; Horridge, 2009). In this cuttlefish study, the black and white silhouettes consist of the same area and both have a high-contrast edge; the difference is that the images have opposite contrast polarity. Interestingly the animals responded differently to the two types of images. This suggests that contrast polarity of a silhouette is a crucial cue during objection recognition. Black and white silhouettes from a biological perspective are related to two natural circumstances under which such high contrast is likely to be perceived. These are a shadow against a background light source and an object glowing in the dark, respectively. Cuttlefish perhaps view an images consisting of a black patch in the shape of prey on a white background as the silhouette of prey when they are looking upward in water toward the sun. On the other hand, an image involving a white patch on a black background might be prey with an extraordinarily high bioluminescence. The former is likely to be much more common in the cuttlefish's natural environment and this perhaps explains the animal's better visual equivalence in our study when it meets the former stimulus.

### Object recognition and visual completion

Amodal completion is a cognitive ability in animals whereby the viewing of a partially occluded object is treated by the animal as the entire entity; this is particularly important when detecting prey or predators. For instance, in the complex structures such as coral reefs, the visual stimuli that invoke territorial behavior in the coral reef fish may be a fragmented one (Darmaillacq et al., 2011). Darmaillacq et al. showed that two species of reef fishes, *Variola louti* and *Scarus niger*, exhibited territorial behaviors toward arrays of mirrors by responding as if they recognized an intruder. In another field experiment, two species of tits, *Poecile palustris* and *Poecile montanus*, tended to keep away from the partially occluded dummy of their natural enemies (Tvardíková and Fuchs, 2010). Our results also support the hypothesis that cuttlefish are able to complete a fragmented image of prey amodally. However, alternatively, all the experiments described above can also be interpreted as the outcome of recognizing specific bodily features rather than amodal completion of the image. The fact that there was different performances by the cuttlefish when the tasks involved half-body occluded images of either the front or back of the prey implies that the anterior part of the body may be more important to amodal completion than the posterior part or, alternatively, the critical features needed for recognition are located in the anterior part of prey. The presence of these specific features may influence the outcome of amodal completion. Thus we suggest that amodal completion leading to the image entity that is related to the original images may depend on the successful recognition of one or perhaps more key features.

In previous studies the ability to carry out contour completion by cuttlefish (*S. officinalis*) via their innate behavior, namely camouflage body patterning, was examined (Zylinski et al., 2009, 2012). Cuttlefish were found to respond to either full circles or fragmented circles with similar disruptive patterns, but showed a different body pattern in response to the rotated and scattered fragments (Zylinski et al., 2012). This result suggests that cuttlefish are able to complete the broken circles and recognize them as whole objects, whereas rotated and scattered fragments are interpreted as small and individual objects in the scene. It also supports that cuttlefish can reconstruct fragmented information and perform modal completion when presented with incomplete boundary information.

### Individual difference exists regarding visual processing by cuttlefish

In the present study, we found that the performance of individual cuttlefish with each task varied somewhat and there is no general way of distinguishing the degree of difficulty of a given task with respect to an individual animal. That is, although all cuttlefish seem to be equipped with the ability of visual equivalence, the performance regarding this ability seems to vary quite a lot. This may be a universal phenomenon across all animal cognition. Previous studies of cephalopod behavior have also provided evidence of individual differences (Darmaillacq et al., 2014). One example is that each individual cuttlefish has a specific side-turning preference and another is that they employ one of the two strategies, response learning or place learning, during a spatial learning paradigm (Alves et al., 2007). Performance differences between individual animals have also been observed during a conditional discrimination test (Hvorecny et al., 2007). Furthermore, episodic personality has been found in gloomy octopuses (*Octopus tetricus*) in a playback study (Pronk et al., 2010). These octopuses could either behave in a shy or bold manner consistently across different experimental contexts over the same day, but this personality trait was not repeatable over a longer time, that is multiple days. Taken together, these findings support that individual variations observed in the present study may result from individual differences in their visual processing abilities.

## Ethics statement

The animal subjects used in the present study are cuttlefish, which are invertebrates and are exempt from this requirement.

## Author contributions

IL conceived, designed, carried out the work, and drafted the manuscript. CC helped plan experiments, interpreted data, and revised the manuscript.

### Conflict of interest statement

The authors declare that the research was conducted in the absence of any commercial or financial relationships that could be construed as a potential conflict of interest.
